# Liver Fibrosis and Hearing Loss in an Older Mediterranean Population: Results from the Salus in Apulia Study

**DOI:** 10.3390/jcm11237213

**Published:** 2022-12-05

**Authors:** Rossella Tatoli, Sarah Tirelli, Luisa Lampignano, Fabio Castellana, Ilaria Bortone, Roberta Zupo, Giancarlo Sborgia, Madia Lozupone, Francesco Panza, Gianluigi Giannelli, Nicola Quaranta, Heiner Boeing, Rodolfo Sardone

**Affiliations:** 1 Unit of Data Sciences and Technology Innovation for Population Health, Department of Basic Medicine, National Institute of Gastroenterology “Saverio de Bellis”, Research Hospital, 70013 Castellana Grotte, Italy; 2 Neurodegenerative Disease Unit, Department of Basic Medicine, Neuroscience, and Sense Organs, University of Bari Aldo Moro, 70013 Bari, Italy; 3 Scientific Direction of National Institute of Gastroenterology “Saverio de Bellis”, Research Hospital, 70013 Castellana Grotte, Italy; 4 German Institute of Human Nutrition Potsdam-Rehbruecke (DIfE), Arthur-Scheunert-Allee 114-116, 14558 Nuthetal, Germany

**Keywords:** liver fibrosis, hearing loss, older Mediterranean population, inflammation, oxidative stress

## Abstract

Background: Aging is the main negative prognostic factor for various chronic diseases, such as liver fibrosis, and clinical disorders such as hearing loss. This study aimed to investigate the association between age-related hearing loss (ARHL) and age-related central auditory processing disorder (CAPD), and the risk for liver fibrosis in a cross-sectional study on an aging population. Methods: Liver fibrosis risk was judged on the fibrosis-4 (FIB-4) score. Peripheral ARHL was evaluated with pure tone audiometry using a calibrated audiometer. The pure tone average (PTA), calculated as a threshold ≤ 40 dB (HL) in the better ear, was measured at the frequencies 0.5–4 kHz. For age-related CAPD assessment, we employed the Synthetic Sentence Identification with an Ipsilateral Competitive Message test (SSI-ICM). General linear Logistic regression models were used to estimate the association. Results: The increase in the PTA 0.5–2 kHz (coefficient: 0.02, SE: 0.01, CI 95%: 0.01 to 0.03) was directly associated with a higher risk of liver fibrosis (FIB-4 ≥ 2.67). Moreover, the reduction in SSI (coefficient: −0.02, SE: 0.01, CI 95%: −0.03 to −0.01) was inversely associated with FIB-4 values < 2.67. Conclusion: Our results show an association between liver fibrosis and both ARHL and CAPD, linked by the typical consequence of aging. We also assume a role of inflammatory responses and oxidative stress.

## 1. Introduction

Liver fibrosis results from several insults leading to the death of hepatocytes and the stimulation of fibrogenesis [1]. It develops after chronic liver injury due to various factors, such as alcohol consumption, nonalcoholic steatohepatitis (NASH), viral hepatitis (hepatitis B and hepatitis C), autoimmune hepatitis, nonalcoholic fatty liver disease (NAFLD), and cholestatic liver diseases. Aging is a major risk factor for most chronic diseases and for liver diseases. It is an adverse prognostic factor [2] and increases the susceptibility to clinical disorders such as visual disturbance, hearing loss, and dementia [3]. Aging negatively affects the structure and function of the liver and is associated with a gradual alteration in hepatic cells and an expansion in neutral fat and cholesterol volumes [3]. Some previous studies suggested that aging is a susceptibility factor for liver fibrosis [4]. The metabolic changes in advanced age increase the prevalence and the risk factors for liver diseases in older populations as compared to the general public [5]. Aging-related changes include increased oxidative stress and inflammatory responses, accelerated cellular senescence, progressive organ dysfunction, and a decreased regenerative ability, which significantly delays the restoration of liver function [6].

Inflammation, or the activation of innate immune responses, is a common pathway through which early life injury can cause long-term chronic disease in peripheral and central neural tissues [7]. With several multifactorial conditions, chronic inflammatory processes also contribute to many sensorial dysfunctions, including presbycusis, the most common sensory damage in aging. Some studies demonstrated that inflammatory processes affecting the hearing system, particularly the cochlea, have a role in the development of age-related hearing loss (ARHL). The effects of these processes, along with lifestyle and dietary factors, are associated with hearing loss in older people [8]. These factors contribute to cardiovascular disease risk, which promotes inflammatory processes in the cochlea and, consequently, hearing dysfunction as an indirect consequence of cardiovascular disease [9].

ARHL is characterized by a form of peripheral ARHL and age-related central auditory processing disorder (CAPD). Peripheral ARHL occurs with changes in inner ear structures, primarily with a progressive decline in cochlear functions and elevated hearing threshold. Instead, age-related CAPD is a disorder of the central auditory pathway. This mechanism reduces abilities to understand speech against background noise or competitive speech and is not involved in peripheral ARHL [10].

This study aimed to investigate the association between different types of age-related hearing loss and the risk for liver fibrosis in a cross-sectional study on an aging population from a Mediterranean area. To the best of our knowledge, no other study has investigated these aspects in older Italian populations.

## 2. Materials and Methods

### 2.1. Study Population and Design

This study involved 1929 subjects aged over 64 years old, representing a subsample from the “Salus in Apulia Study” (*n* = 4,537), enrolled from 2014 to 2019 in Castellana Grotte, Southern Italy. The sample was representative of the entire population of older people (age > 65 years) of Castellana Grotte in 2014, as described elsewhere [11]. This sample included subjects that underwent clinical, lifestyle, and hearing assessments. Subjects with missing audiometry or an inability to attend to the required tests, diseases of the middle ear, and congenital deafness were excluded. All participants signed informed consent forms, and the study was approved in 2014 and again in 2019 by the IRB of the National Institute of Gastroenterology “S. De Bellis”, where all the examinations described in this study were performed. The present study adhered to the “Standards for Reporting Diagnostic Accuracy Studies” (STARD) guidelines (http://www.stard-statement.org/ accessed on 3 September 2022), the “Strengthening the Reporting of Observational Studies in Epidemiology” (STROBE) guidelines https://www.strobe-statement.org/ accessed on 3 September 2022), and followed the Helsinki Declaration of 1975.

### 2.2. Clinical and Lifestyle Assessment

In the present study, the evaluation of lifestyle and anthropometric parameters was entrusted to a physician during an interview. Body mass index (BMI) was calculated as kg/m^2^. Height and weight measurements were performed using a Seca 220 stadiometer and a Seca 711 scale. Systolic blood pressure (SBP) and diastolic blood pressure (DBP) were determined in a sitting position after at least a 10 min rest and at least three different times using the OMRON M6 automatic blood pressure monitor. A blood sample was collected in the morning after overnight fasting to measure the levels of fasting blood glucose (FBG), glycated hemoglobin (HbA1c), total cholesterol, transaminases, and triglycerides using standard automated enzymatic colorimetric methods (AutoMate 2550; Beckmann Coulter, Brea, CA, USA) under strict quality control. The platelet count was determined with a Coulter Hematology analyzer (Beckman Coulter, Brea, CA, USA). The level of education was expressed as years of schooling.

### 2.3. Fibrosis-4 Index for Liver Fibrosis Risk (FIB-4)

The FIB-4 is one of the noninvasive methods studied for diagnosing liver fibrosis [12]. It is simple to use and quick to calculate because it combines standard biochemical values (platelets, ALT, AST) and age [13].

The FIB-4 index enables the correct identification of extreme types of fibrosis because its performance seems to be reliable in identifying very moderate fibrosis versus more severe forms of the disease [14]. In one study comparing FIB-4 to other noninvasive fibrosis markers, FIB-4 most correctly identified F3–F4 fibrosis with values more than 2.67 (positive predictive value 80%) in biopsy-proven NAFLD patients. Using this cutoff, advanced fibrosis was identified with 89% accuracy [15].

The use of simple noninvasive fibrosis scores such as FIB-4 to classify or exclude advanced fibrosis as part of a step-by-step approach to diagnosis and risk stratification in patients with NAFLD is generally followed and recommended in most guidelines [16,17].

### 2.4. Hearing Assessment

The hearing functions were assessed in all participants by a certified audiologist. An otoscopy and tympanometry (Interacoustics Audio Traveller AA222; Audiometer Alle 1, 5500 Middelfart, Danimarca ) were conducted to rule out middle and external ear dysfunction that could induce conductive hearing loss. The pure-tone audiometry procedure was performed as a measurement of the hearing threshold, according to the Hughson–Westlake method, with a calibrated audiometer (Interacoustics Affinity Compact with AC440 modules) and Radioear DD65 v2 headphones (Sennheiser Electronic; GmbH & Co. KG, Wedemark, Germany) in a soundproof room. We calculated the pure tone average (PTA) as an indicator of peripheral ARHL on the frequencies of 500, 1000, and 2000 Hz [18]. Only the participants with a PTA ≤ 40 dB HL in the better ear underwent the Italian version of the Synthetic Sentence Identification–Ipsilateral Competing Message (SSI-ICM) test. [19]. This competitive test is a sensitive, specific measure to diagnose age-related CAPD. The SSI-ICM consists of administering, for each ear, a primary signal of ten brief sentences against a contextual competition signal. The test scoring is expressed as a percentage (0–100%), and normal subjects reach 100% identification. In accordance with Gates [20] and Sardone [21], a central auditory speech-processing deficit (CAPD) was defined as a score of ≤50% in at least one ear.

### 2.5. Statistical Analyses

From the whole sample, two groups were identified, according to the FIB-4 cut-off (2.67), to describe clinical and functional differences in frequency and associations. The normality of variables distribution was tested using a Shapiro–Wilk test.

Because the variables were not normally distributed, continuous variables were expressed as the median (with relative full ranges and interquartile ranges (IQR)), and categorical variables were expressed as a number (percentage). A no-parametric approach was followed, and Wilcoxon’s effect size was adopted to evaluate the magnitude of the differences between groups for continuous variables. Differences in proportions were assessed using prevalence differences between groups. The interpretation values and commonly selected thresholds for Wilcoxon’s ES are: small magnitude of differences (ES < 0.10), moderate magnitude of differences (0.10 ≤ ES < 0.30), and large magnitude of differences (ES ≥ 0.50). The sign of the ES showed the direction of the difference according to the comparison groups; if positive, values in the first group was higher than in the second one. Tomczak M and Tomczak E. revisited the need to report effect size estimates and provided an overview of some recommended measures of effect size [22]. Considering the non-normal distribution and possible nonlinear associations between the variables of interest, rank-based estimators were used instead of standard generalized linear models. Rank-based regression is a robust, nonparametric alternative to the traditional likelihood or least squares estimators [23]. Three different nested models were built for each hearing-dependent variable (PTA and SSI-ICM), hierarchically adjusted for a set of confounders by three different levels (Model 1: unadjusted; Model 2: adjusted for constitutional confounders: gender, BMI, and educational level; Model 3: adjusted for all confounders, age, gender, BMI, education, HbA1c, total cholesterol, triglycerides, and DPB). All the confounders, i.e., gender, BMI, educational level, HbA1c, total cholesterol, triglycerides, and DPB, were chosen based on their probable biological association with both the exposure (hearing loss) and the outcomes (Fib-4) [24]. All statistical analyses were performed using RStudio 22.02.3.

## 3. Results

The average age of the sample was 74.6 ± 6.25 years, and the male gender was predominant (*n* = 974; 53.5%).

The participants were divided into two groups according to the FIB-4 cut-off (2.67).

Table 1 shows the main sociodemographic and clinical characteristics of the older subjects involved in the study.

In the whole sample, 435 patients had a FIB-4 ≥ 2.67 score; they were predominantly male (*n* = 256; 58.9%; E.S.: −10.79 (−16.06 to −5.52)), older (76.77 ± 6.61; E.S.: 0.26 (0.22 to 0.31)), and had a lower level of education (6.32 ± 3.56; E.S.: 0.09 (0.05 to 0.14)) than the members of the other group (FIB-4 < 2.67).

The group with a FIB-4 ≥ 2.67 had a higher percentage of subjects with ARHL (28.7% vs 20.1%; E.S.: 8.66 (3.94 to 13.37)) and CAPD (18.6% vs 13.4%; E.S.: 4.05 (0.07 to 8.04)). Consequently, they had a higher value of PTA 0.5-2kHz (32.60 ± 13.80 vs 29.80 ± 12.00; E.S.: 0.20 (0.15 to 0.24)).

Table 2 and Table 3 show rank regression models on the FIB-4 score as a dependent score and lower PTA 0.5–2 kHz and lower SSI as regressors, respectively. The increase in the PTA 0.5–2 kHz (coefficient: 0.02, SE: 0.01, CI 95%: 0.01 to 0.03) was directly associated with a higher risk of liver fibrosis (FIB-4 ≥ 2.67). Moreover, the reduction in SSI (coefficient: −0.02, SE: 0.01, CI 95%: −0.03 to −0.01) was inversely associated with the values of FIB-4 < 2.67. These associations were also maintained in the partially adjusted model and fully adjusted model.

## 4. Discussion

In this study, we found a significant association between the risk of liver fibrosis, assessed with the FIB-4 score, and age-related hearing impairment, among a cohort of the older population of the Mediterranean area.

Age is a common risk factor for both liver fibrosis and hearing loss. Some studies suggested that aging negatively influences liver function through a substantial morphological change in the sinusoidal vascular system [25], while little is known about aging effects on liver sinusoidal endothelial cells, Kupffer cells, and hepatic stellate cells [3]. ARHL has multifactorial pathogenesis and is an inevitable hearing impairment related to aging. The reduction in hearing ability is due in particular to physiological factors and structural changes typical of the elderly, such as the degeneration of the cochlea, but also to intrinsic disorders such as systemic diseases [26]. Some studies reported several risk factors contributing to hearing loss, including genetic factors, inflammatory processes, systemic diseases, oxidative stress, and aging [27,28].

Liver fibrosis is often also the result of advanced NAFLD liver injury [29]. The prevalence of NAFLD associated fibrosis has surged, showing more than a twofold increase over the past two decades [30].

Lately, NAFLD has emerged as the most common cause of chronic liver disease in developed countries and affects an estimated 25% of the global population [31]. NAFLD can be considered more than just a manifestation of Metabolic Syndrome (MetS) because recent studies have demonstrated that it increases the incident risk of MetS, diabetes, and cardiovascular diseases [32]. NAFLD has also been associated with obesity, diabetes mellitus, hypertension, and dyslipidemia [33,34].

Our results seem to conflict with this evidence because the levels of fasting blood glucose (FBG), glycated hemoglobin (HbA1c), total cholesterol, triglycerides, systolic blood pressure (SBP), and diastolic blood pressure (DBP) were similar or lower in the group of subjects with a FIB-4 ≥ 2.67 than in the other group. Two hypotheses could explain these results: firstly, aging is associated with a physiological reduction in blood pressure and several biochemical markers [35]. Secondly, the use of specific pharmacologic therapy (e.g., antihypertensives, statins, and diabetes drugs) by the subjects with a FIB-4 ≥ 2.67 may have affected the results. Some studies have demonstrated the relationship between hearing loss and metabolic inflammatory diseases, such as diabetes and cardiovascular diseases [9,36]. This association has also been demonstrated for other disease characteristic of MetS, such as hypertension and dyslipidemia [37]. This evidence could indirectly support our results because liver fibrosis, in some cases, is a consequence of NAFLD, which fits into the framework of MetS.

Inflammation could link liver fibrosis and hearing loss in older people.

Inflammation is a complex process. It is an instrument by which an organ or tissue responds to a local insult in order to restore the original structure and function. Several studies showed that hepatic and systemic injury is related to the high production of pro-inflammatory cytokines [38]. These cytokines, produced in various organs after tissue damage, cause or accelerate long-term damage to the auditory system with aging. It has been found that cytokines produced by the cochlear structure itself, such as IL-6, which are present in fibrocytes in the stria vascularis and spiral ganglion neurons, may trigger an inflammatory response and have some role in the mechanism of cochlear damage [39]. Several studies have reported the existence of inflammatory cells in the steady state and their increase after damage to the inner ear [40,41,42]. One explanation would be a local upregulation of proinflammatory cytokines because these molecules are endogenously elevated in the early stages in the damaged cochlear (within one day) and generally induce the infiltration of inflammatory cells, which increases in the cochlea up to maximum levels 3–7 days after the damage [43].

IL-6 is one of the main cytokines involved in inflammation. Various cell types produce them during tissue damage, infection, and inflammatory diseases [44]. So et al. observed the transient upregulation of IL-6 in cisplatin-treated models, a common damaged cochlea model and one with noise-over stimulation [45]. It is also known that the upregulation of IL-6 and subsequent STAT3 regulatory cascades promote hepatic stellate cell (HSC) activation, causing liver fibrosis and cirrhosis [46]. IL-6 could lead to excessive inflammatory activation that induces oxidative stress, followed by tissue damage and liver disease progression [47]. The activated HSC creates a vicious circle in the process of liver fibrosis, known as the inflammation-fibrosis axis [48]. In our sample, IL-6 levels were higher in the group with FIB ≥ 2.67.

Increased oxidative stress and inflammatory responses, with a consequent reduction in the cellular ability to respond to injury, are typical age-related changes [6]. Some studies have investigated the association of age-dependent liver injury and fibrosis with the immune milieu [4,49]. These suggest that the main factor involved in the greater susceptibility to fibrosis with aging is an increased inflammatory reaction, mainly composed of CD4(+) lymphocytes and macrophages expressing helper T cell type 2 cytokines [4].

### Strengths and Limitations

Our study investigated the association between liver fibrosis and hearing loss in older Mediterranean people. This is its main strength because, to our knowledge, no study has analyzed these aspects in similar populations. Another important strength includes its large sample size and the possibility to generalize the results to an older Southern Mediterranean population. For the hearing evaluation, a complete and in-depth audiological assessment was made, which is another important strength of this study. A limitation is represented by the information on liver fibrosis status because the data detected by transient elastography (FibroScan) or biopsy and MRI (Magnetic Resonance Imaging) were not available. To address this limitation, we used the FIB-4 score as a surrogate because this noninvasive score is recommended by several guidelines [16,17]. Another limitation is the absence of data on medication in our population.

## 5. Conclusions

In conclusion, the present study draws attention to the link between liver fibrosis and hearing loss. Typical age-related changes unite these two conditions. A role could also be played by an increase in oxidative stress and inflammatory responses. However, further studies are warranted in this regard because, in the scientific literature, there are currently no studies evaluating these aspects. Future research could also address whether environmental factors (for example, diet, smoking, and lifestyle) are involved in slowing or accelerating the onset of these conditions in the older population.

## Figures and Tables

**Table 1:** Description of the whole sample according to FIB-4 cutoff (<2.67/≥2.67).

	FIB < 2.67 (*n* = 1494; 77.4%)		FIB ≥ 2.67 (*n* = 435; 22.6%)		
	Mean ± Sd	Median (Min to Max)	Mean ± Sd	Median (Min to Max)	Effect Size
Age (years)	72.62 ± 5.89	71 (65 to 95)	76.77 ± 6.61	77 (65 to 95)	0.26 (0.22 to 0.31)
Sex					
Male	718 (48.10)		256 (58.90)		−10.79 (−16.06 to −5.52)
Female	776 (51.90)		179 (41.10)		
Educational Level (years)	7.12 ± 3.88	5 (0 to 23)	6.32 ± 3.56	5 (0 to 18)	0.09 (0.05 to 0.14)
Diastolic Blood Pressure (DBP) (mmHg)	78.39 ± 7.62	80 (50 to 110)	77.03 ± 8.86	80 (40 to 100)	0.07 (0.03 to 0.12)
Systolic Blood Pressure (SBP) (mmHg)	132.84 ± 14.38	130 (80 to 200)	133.79 ± 14.87	130 (100 to 180)	0.02 (−0.02 to 0.06)
Fasting Blood Glucose (FBG) (mg/dL)	105.66 ± 28.55	99 (54 to 435)	106.54 ± 27.7	100 (66 to 365)	0.02 (−0.02 to 0.06)
Glycated Hemoglobin (HbA1c) (mmol/mol)	40.43 ± 10.6	39 (19 to 128)	40.76 ± 10.24	39 (18 to 101)	0.01 (−0.02 to 0.05)
GGt	29.85 ± 32.42	18 (5 to 158)	46.52 ± 44.01	27 (6 to 158)	0.19 (0.15 to 0.24)
GOT	24.21 ± 9.9	22 (1.2 to 189)	57.17 ± 46.12	41 (16 to 197)	0.51 (0.48 to 0.55)
GPT	25.25 ± 18.84	19 (7 to 221)	27.45 ± 25.32	18 (4 to 180)	0.03 (−0.01 to 0.08)
Total Cholesterol (mg/dL)	186.4 ± 37.34	186 (76 to 386)	174.2 ± 35.29	175 (76 to 278)	0.13 (0.09 to 0.18)
Triglycerides (mg/dL)	108.92 ± 61.12	95 (21 to 773)	97.88 ± 60.66	84 (17 to 773)	0.10 (0.06 to 0.15)
IL6 (pg/mL)	3.76 ± 6.3	1.89 (0.06 to 64.94)	4.57 ± 7.99	2.18 (0.1 to 64.94)	0.07 (0.03 to 0.12)
CRP (mg/dL)	0.59 ± 0.89	0.32 (0.1 to 10.96)	0.59 ± 0.74	0.33 (0.1 to 6.6)	0.01 (−0.02 to 0.04)
Platelets (103 cells/mm³)	233.99 ± 57.72	226 (104 to 832)	184.08 ± 53.58	180 (61 to 459)	0.36 (0.33 to 0.41)
Central Auditory Processing Disorder (CAPD)	200 (13.40)		81 (18.60)		4.05 (0.07 to 8.04)
Age-Related Hearing Loss (ARHL)	300 (20.10)		125 (28.70)		8.66 (3.94 to 13.37)
Pure Tone Average (PTA) 0.5–2 kHz (Worst ear)	29.80 ± 12.00	25 (5 to 90)	32.60 ± 13.80	30 (2 to 100)	0.20 (0.15 to 0.24)
Synthetic Sentence Identification (SSI) (lower)Identification (SSI)	63.11 ± 36.6	70 (0 to 100)	50.90 ± 36–20	60 (0 to 100)	0.15 (0.11 to 0.19)

All data are shown as mean ± sd, median (min to max) for continuous variables, and as *n* (%) for proportions.

**Table 2:** Multiple rank-based regression models of FIB-4 score as dependent variable.

	Model 1			Model 2			Model 3		
	Coefficient	Stand. Err.	CI 95%	Coefficient	Stand. Err.	CI 95%	Coefficient	Stand. Err.	CI 95%
PTA 0.5–2 kHz (Worst ear)	0.02	0.02	0.01 to 0.03	0.02	0.01	0.01 to 0.03	0.02	0.02	0.01 to 0.03
Sex (Female)				−0.23	0.04	−0.3 to −0.16	−0.22	0.04	−0.29 to −0.14
BMI (Kg/m^2^)				0.01	0.01	−0.01 to 0.02	0.01	0.01	−0.01 to 0.02
Educational Level				−0.02	0.01	−0.03 to −0.01	−0.02	0.02	−0.03 to −0.01
HbA1c (mmol/mol)							0.01	0.02	−0.02 to 0.03
Total Cholesterol (mg/dL)							−0.02	0.01	−0.03 to −0.01
Triglycerides (mg/dL)							−0.02	0.02	−0.03 to −0.01
DBP (mmHg)							−0.02	0.03	−0.03 to −0.01

**Table 3:** Multiple rank-based regression models of FIB-4 score as dependent variable.

	Model 1			Model 2			Model 3		
	Coefficient	Stand. Err.	CI 95%	Coefficient	Stand. Err.	CI 95%	Coefficient	Stand. Err.	CI 95%
SSI (Worst ear)	−0.02	0.02	−0.03 to −0.01	−0.02	0.02	−0.03 to −0.01	−0.02	0.02	−0.03 to −0.01
Sex (Female)				−0.22	0.04	−0.29 to −0.15	−0.21	0.04	−0.29 to −0.14
BMI (Kg/m^2^)				−0.01	0.02	−0.02 to 0.02	0.01	0.02	−0.01 to 0.03
Educational Level (years)				−0.02	0.01	−0.03 to −0.01	−0.02	0.01	−0.03 to −0.01
HbA1c (mmol/mol)							0.01	0.02	−0.02 to 0.03
Total Cholesterol (mg/dL)							−0.02	0.01	−0.03 to −0.01
Triglycerides (mg/dL)							−0.02	0.02	−0.03 to −0.01
DBP (mmHg)							−0.02	0.01	−0.03 to −0.01
SSI (Worst ear)	−0.02		−0.03 to −0.01	−0.02	0.01	−0.03 to −0.01	−0.02	0.01	−0.03 to −0.01

Model 1: unadjusted. Model 2: adjusted for constitutional confounders sex, BMI, and education. Model 3: adjusted for all confounders, i.e., age, sex, BMI, education, HbA1c, total cholesterol, triglycerides, and DPB. SSI: Synthetic Sentence Identification. PTA 0.5–2 kHz: pure tone average.

## Data Availability

The datasets used and/or analyzed during the current study are available from the corresponding author upon reasonable request.

## Abbreviations

ARHL:	Age-Related Hearing Loss
BMI:	Body Mass Index
CAPD:	Central Auditory Processing Disorder
DBP:	Diastolic Blood Pressure
FBG:	Fasting Blood Glucose
FIB-4:	Fibrosis-4 Index for Liver Fibrosis Risk
HbA1c:	Glycated Hemoglobin
HSC:	Hepatic Stellate Cell
MetS:	Metabolic Syndrome
MRI:	Magnetic Resonance Imaging
NASH:	Nonalcoholic Steatohepatitis
NAFLD:	Nonalcoholic Fatty Liver Disease
PTA:	Pure Tone Average
SBP:	Systolic Blood Pressure
